# Supplementary material for: Association between tuberculosis and depression on negative outcomes of tuberculosis treatment: A systematic review and meta-analysis
Source: PLoS One. 2020 Jan 10;15(1):e0227472. doi: 10.1371/journal.pone.0227472 (PMC6953784; doi:10.1371/journal.pone.0227472)
Supplement: S1 Table — (DOCX) [file pone.0227472.s002.docx]

**S1 Table. Search strategy for other databases.**

| **Database** | **Search strategy** |
| --- | --- |
| EMBASE | ´tuberculosis´:ti,ab,kw AND ´depression´:ti,ab,kw AND [humans]/lim AND ([embase]/lim OR [embase classic]/lim OR [pubmed-not-medline]/lim) |
| Scopus | TITLE-ABS-KEY (tuberculosis AND depression) |
| Web of Science | TS=(tuberculosis AND depression) OR TI=(tuberculosis AND depression) |
| Global Health Library | tuberculosis AND depression |
