# Supplementary material for: Association between tuberculosis and depression on negative outcomes of tuberculosis treatment: A systematic review and meta-analysis
Source: PLoS One. 2020 Jan 10;15(1):e0227472. doi: 10.1371/journal.pone.0227472 (PMC6953784; doi:10.1371/journal.pone.0227472)
Supplement: Attachment — Submitted filename: Response to Reviewers.docx [file pone.0227472.s003.docx]

Dear Prof. Hasnain Seyed Ehtesham

Academic Editor

PLOS One

Thank you for reviewing our manuscript entitled “Association between Tuberculosis and Depression on Negative Outcomes of Tuberculosis Treatment: A Systematic Review” by Ruiz-Grosso et al. for publication as a research article in PLOS ONE. We have received your comments and have corrected the manuscript accordingly. Please find the response to the comments made bellow:

1. **Please ensure that your manuscript meets PLOS ONE’s style requirements, including those for file naming.**

Changes have been made to the manuscript’s style to fulfill PLOS ONE’s requirements, including headings, figure and table citations, and file naming.

2. **Please include captions for your Supporting Information files at the end of the manuscript and update any in-text citations to match accordingly.**

Appropriate captions and in-text citations have been made for Supporting Information. A separate file has been created for S1 Table and has been submitted.

3. **Funding information should not appear in the Acknowledgments section. Please remove any funding-related text from the manuscript and let us know how you would like to update your Funding Statement section of the online submission form.**

Funding-related text has been removed from the manuscript. Please update the Funding Statement section of the online submission form to read: “PRG had support from FONDECYT/CIENCIACTIVA scholarship EF033-235-2015 and from training grant D43TW007393 awarded by the Fogarty International Center of the US National Institutes of Health. The authors had full access to the study’s data and were responsible for the decision to submit for publication.”

4. **Authors have been extremely careless with their citations. These must be fixed and extensively updated.**

References have been added when addressing the papers included in the systematic review. Furthermore, citations have been revised to make sure they are in the correct order and contain the information that we claim in the discussion.

5. **Authors should expand on DOTS. This would help identify to the reader what non-adherence means.**

Two short sentences have been added in introduction which briefly describe DOTS program to help readers understand the usual outpatient setting and program employed to ensure adherence as a public health measure.

6. **Studies to show how much non-adherence is acceptable.**

We have expanded the discussion on the definition and possible significance of non-adherence during TB treatment. We wish to transmit the message that there is no standardized definition for non-adherence and there is uncertainty regarding the number of missed doses needed to alter the treatment outcome. Thus, there is no level of acceptable non-adherence, however, some of the included studies do find an important association between non-adherence as defined per study. This is discussed in page 9, line 160-166, where we describe the ways non-adherence was defined in each study. Furthermore, in page 14, line 240-246, we consider this as an important limitation for the study.

7. **In Page 2 Line 36: State that MDR-TB is multidrug-resistant tuberculosis when the term is first introduced.**

MDR-TB is first introduced as multidrug-resistant TB.

8. **Reference 7 appears to be incorrect. Double check the figure.**

Reference has been updated to WHO Global Health Estimates for total number of people living with depression.

9. **Authors must find a better reference than reference 8 to support their claims.**

Reference has been updated to WHO Global Health Estimates for prevalence of depression among males and females.

10. **Reference 12 appears to be a circular reference.**

Reference has been updated to cite the actual research article that supports our claims.

11. **In Page 9 Line 198: State that “depressive symptoms” is DS and “psychological distress” is PD.**

“Depressive symptoms” and “psychological distress” are referenced as DS and PD in page 13, line 210-211, respectively.

12. **Comment on the age of the study participants that they used for the meta-analyses; especially for death as an outcome.**

Information on age has been added to Table 2 and 3 which report characteristics and summary of findings of the included articles. This has also been addressed in page 14, line 251-262, where we specifically mention age in relation to the analysis that evaluates the association with death as an outcome.

13. **Address the heterogeneity in the studies including the definition of depression and treatment outcomes.**

We have reviewed the discussion regarding the heterogeneity on the definition of both the exposure (depression and psychological distress) and outcome (death, loss to follow up and non-adherence) which can be found on page 13-14, line 223-249. Depression was measured using three different scales (CES-D in full and short versions, PHQ-9), however, the scales were validated to detect severe forms of depression which might explain the low heterogeneity on the main meta-analysis results. PD was measured using the K-10 scale, albeit with different cut-off scores. Death and loss to follow up had clear definitions throughout the studies, while the variability of definitions for non-adherence has been addressed above in point 6.

We hope you find our responses to your comments to be appropriate. We look forward to hearing from you at your earliest convenience.

Yours sincerely,

César Ugarte-Gil

Corresponding Author

Instituto de Medicina Tropical Alexander von Humboldt

Universidad Peruana Cayetano Heredia
